# Trends in cancer of the cervix uteri in Sweden following cytological screening

**DOI:** 10.1038/sj.bjc.6690666

**Published:** 1999-09

**Authors:** R Bergström, P Sparén, H-O Adami

**Affiliations:** Department of Information Sciences, Division of Statistics, PO Box 513, Uppsala, SE-751 20, Sweden; Department of Medical Epidemiology, Karolinska institutet, PO Box 281, Stockholm, SE-171 77, Sweden; Stockholm Centre on Health of Societies in Transition, Södertörns högskola, PO Box 4101, Huddinge, SE-141 04, Sweden; Department of Epidemiology, Harvard School of Public Health, Boston, 02115, USA

**Keywords:** cytological screening, cervical cancer, squamous cell cancer, adenocarcinoma, age-period-cohort models

## Abstract

Trends in cervical cancer incidence following the introduction of screening have mostly been studied using cross-sectional data and not analysed separately for squamous cell cancer and adenocarcinomas. Using Swedish nationwide data on incidence and mortality, we analysed trends during more than 3 decades and fitted Poisson-based age-period-cohort models, and also investigated whether screening has reduced the incidence of adenocarcinomas of the cervix. The incidence of reported cancer in situ increased rapidly during 1958–1967. Incidence rates of squamous cell cancer, fairly stable before 1968, decreased thereafter by 4–6% yearly in women aged 40–64, with a much smaller magnitude in younger and older women. An age-cohort model indicated a stable 70–75% reduction in incidence for women born 1940 and later compared with those born around 1923. The incidence of adenocarcinomas doubled during the 35-year study period. The mortality rate increased by 3.6% before 1968 and decreased by 4.0% yearly thereafter. Although a combination of organized and opportunistic screening can reduce the incidence of squamous cell cancer substantially, the incidence of adenocarcinomas appears uninfluenced by screening. © 1999 Cancer Research Campaign


*British Journal of Cancer* (1999) **81**(1), 159–166
© 1999 Cancer Research Campaign
Article no. bjoc.1999.0666

### Trends in cancer of the cervix uteri in Sweden following cytological screening

**R Bergström[1,2,*] P Sparén[2,3,*] and H-O Adami[2,4]**

[1]Department of Information Sciences, Division of Statistics, Uppsala University, PO Box 513, SE-751 20 Uppsala, Sweden; [2]Department of Medical Epidemiology, Karolinska institutet, PO Box 281, SE-171 77 Stockholm, Sweden; [3]Stockholm Centre on Health of Societies in Transition, Södertörns högskola, University College, PO Box 4101, SE-141 04 Huddinge, Sweden; [4]Department of Epidemiology, Harvard School of Public Health, Boston, 02115- MA, USA

**Summary** Trends in cervical cancer incidence following the introduction of screening have mostly been studied using cross-sectional data and not analysed separately for squamous cell cancer and adenocarcinomas. Using Swedish nationwide data on incidence and mortality, we analysed trends during more than 3 decades and fitted Poisson-based age-period-cohort models, and also investigated whether screening has reduced the incidence of adenocarcinomas of the cervix. The incidence of reported cancer in situ increased rapidly during 1958–1967. Incidence rates of squamous cell cancer, fairly stable before 1968, decreased thereafter by 4–6% yearly in women aged 40–64, with a much smaller magnitude in younger and older women. An age-cohort model indicated a stable 70–75% reduction in incidence for women born 1940 and later compared with those born around 1923. The incidence of adenocarcinomas doubled during the 35-year study period. The mortality rate increased by 3.6% before 1968 and decreased by 4.0% yearly thereafter. Although a combination of organized and opportunistic screening can reduce the incidence of squamous cell cancer substantially, the incidence of adenocarcinomas appears uninfluenced by screening.

**Keywords:** cytological screening; cervical cancer; squamous cell cancer; adenocarcinoma; age-period-cohort models

Cancer of the cervix uteri is the second most common cancer in the world among women and, especially in developing countries, a major cause of premature death in middle-aged and older women (Parkin et al, 1993; Pisani et al, 1993). Cytological screening can reduce morbidity substantially (Hakama, 1982; Pettersson et al, 1985; Anderson et al, 1988; Hakama and Louhivuori, 1988; Gustafsson and Adami, 1990; Levi et al, 1991; Sigurdsson, 1993), although in several populations this benefit was limited, occurred only recently, or could not be demonstrated at all (Villard et al, 1989; Sasieni, 1991; Gustafsson et al, 1997). In the USA, incidence and mortality rates of cervical cancer have been declining for several decades, partly due to PAP-smear screening (Brinton and Fraumeni, 1986; Devesa et al, 1989, 1995). In the UK, mortality rates in younger birth cohorts are heavily reduced, compared to older cohorts (Sasieni et al, 1995). Recent reductions in mortality have been interpreted as benefits from the national screening programme (Farmery and Gray, 1994; Quinn et al, 1999; Sasieni and Adams, 1999). In Sweden, the introduction of population-based screening during the 1960s led to a decline in both incidence and mortality (Pettersson et al, 1985; Gustafsson and Adami, 1990).

The potential of Pap-smear screening to reduce morbidity from cervix cancer has been documented beyond doubt; however, important questions remain to be answered. Most analyses were based on cross-sectional data, i.e. trends in cervix cancer incidence or mortality were studied according to calendar time, while, in reality, the effects of screening are linked to birth cohorts. The reasons for this are threefold: (1) screening is conducted mainly among women of reproductive ages, i.e. only certain birth cohorts are affected at a specific point in time; (2) The positive effects of screening on morbidity and mortality are delayed for 3 years or more; (3) the benefit of removed precursor lesions may continue for at least 1 decade. In Sweden, Pap-smear screening was introduced over a relatively short time period and was offered mainly to young and middle-aged women. Hence, the younger a woman was during the period when PAP smears came into use, the more extensively she is likely to have been screened during the ages at risk of developing cancer of the cervix. Analyses allowing estimation of birth cohort effects should therefore provide more accurate quantitative estimates of effects of screening.

No study has adequately distinguished the effects of screening on the incidence of squamous cell carcinoma and adenocarcinoma of the cervix – and the latter may not be preventable at all by PAP smear screening, since it has no readily detectable preinvasive stage (Kudo, 1992). The incidence of adenocarcinoma appears to be increasing in many populations. Failure to take this trend into account – and, ideally, analyse the incidence of squamous cell cancer separately – may entail underestimation of the benefit of detecting and eliminating cancer in situ.

To clarify these issues, we took advantage of certain features unique to Sweden, including successful nationwide screening programme, accurate cancer incidence and mortality statistics during a period of at least 35 years, and separate registration of

*Received 25 September 1998*
*Revised 1 March 1999*
*Accepted 10 March 1999*

*Correspondence to:* P Sparén, Stockholm Centre on Health of Societies in Transition, Södertörns högskola, University College, PO Box 4101 SE-141 04 Huddinge, Sweden

*Joint first authors.

cancer in situ, squamous cell cancer and adenocarcinoma of the cervix. Our main objectives were: (1) to quantify, by calendar time, age and birth cohort, the overall trends in cervix cancer incidence and mortality and (2) to clarify whether screening has reduced the incidence of adenocarcinoma of the cervix.

### SUBJECTS AND METHODS

### The Cancer Register and the Death Register

Nationwide cancer registration started in Sweden in 1958. According to the regulations, all physicians are required to report all cases of newly diagnosed cancer to the Cancer Registry at the National Board of Health and Welfare. Pathologists and cytologists must also notify the Cancer Registry of every cancer diagnosis made on surgically removed tissues, biopsies, cytological specimens and autopsies. Thus, in the majority of cases, the Registry receives two reports. Cases identified from death certificates alone are not included in the register (National Board of Health and Welfare, 1960–1998). In the 1970s the underreporting of incident cases was estimated at 4.5% and was referable mainly to patients older than 75 years and those with malignant disease of the haematopoietic system (Mattsson and Wallgren, 1984). Reporting is now considered to encompass close to 100% of all diagnosed cases (National Board of Health and Welfare, 1960–1998).

Since 1951, Swedish cause-of-death statistics have been collected and classified according to the International Classification of Diseases (ICD). For each death, a death certificate must be issued by a physician within 1 week. The certificate is forwarded to Statistics Sweden by the local population registers, and demographic information is merged with the information on the death certificate. For each death certificate an underlying cause of death is selected manually. Any deficit in the register is negligible (National Board of Health and Welfare, 1956–1997). Before 1981 a cancer diagnosis was recorded even if it was considered only a contributory cause by the certifying physician. In 1981 the coding routines were changed so that contributory causes of death were no longer included in the cancer mortality statistics.

In the Swedish Cancer Registry, all tumours are coded according to the ICD-7 and according to histopathological type. Cancer of the cervix uteri, squamous cell cancer and adenocarcinomas, as well as adeno-squamous carcinomas, have separate codes in the Registry.

The adeno-squamous carcinomas, a rare histopathological type, were not included in our analyses since their incidence has remained stable during the period of study. The Cancer Registry also requires the reporting of cancer in situ and severe dysplasia, which is on the borderline of cancer in situ (National Board of Health and Welfare, 1984); we refer to these lesions as cancer in situ of the cervix.

Our analysis was based on 110 653 patients diagnosed as having cervix cancer in situ, 21 805 invasive squamous cell cancers and 2584 adenocarcinomas about which the Cancer Registry was notified during the period 1958–1995; and also 10 655 patients who had invasive cervical cancer coded as their cause of death between 1953 and 1995. However, in the age-period cohort modelling (see below) the study period end 1992, since we divided the data in 5-year calendar time periods.

### Screening for cervical cancer

Pap-smear screening was introduced in Sweden firstly on a small scale with testing of possibly symptomatic women. In 1963 about 200 000 smears were taken annually, while in 1970 the figure had increased to about 1 000 000 (National Board of Health and Welfare, 1982). Population-based screening programmes, where women were actively invited for screening, were introduced between 1967 and 1973, except for the city of Gothenburg where an organized screening programme was not begun until 1977 (National Board of Health and Welfare, 1976). All women aged 30–49 years were then invited to undergo an examination at 4-year intervals. Later on, women 25 years of age or more were also invited to participate and the screening interval was shortened to 3 years, although this policy varied somewhat with time and place. When screening was fully implemented in Sweden around one-quarter of the smears were taken in the organized screening programmes, and the remaining three-quarters were taken at hospitals and outpatient clinics through opportunistic testing (National Board of Health and Welfare, 1982; Pettersson et al, 1985; Gustafsson et al, 1995*b*).

### Statistical methods

Incidence and mortality rates were standardized to the Swedish census population in 1970 (National Board of Health and Welfare, 1960–1998). We performed simple trend-analyses for 5-year age-specific rates, as well as for age-standardized rates. We used a model that implied a constant annual relative change in rates by regressing logarithmic rates on linear trend variables. Such trend analyses were also performed for subperiods to allow for changes in growth rates. For simplicity we used the same cut-off year (1968) in these analyses, although this need not be absolutely optimal in all instances. Models including second-order trend terms were also estimated to accommodate non-linear effects and to test the assumption of the basic linear model.

We based age-period cohort analyses of incidence rates on grouped 5-year data comprising 13 age-classes (20–24, …, 80–84 years) and seven time periods when the cancers were diagnosed (1958–1962, …, 1988–1992), which consequently meant 19 partially overlapping birth cohorts (1874–1882, 1879–1887, …, 1964–1972). Correspondingly, mortality rates were analysed from 1953–1957 to 1988–1992, with 20 partially overlapping birth cohorts. To obtain the effects of age, period and cohort on cancer incidence and mortality, models were fitted on the assumption that the number of cases constituted a variable with a Poisson distribution. The effects of age, period and cohort were assumed to be multiplicative, and the parameters of the models were estimated by means of the maximum likelihood method using generalized linear models. Results are presented as relative risks (RR) with 95% confidence intervals (CI). To judge the plausibility of the assumption that the relative differences between birth cohorts are the same at different ages we also estimated separate age-cohort models for various broader age-classes where needed.

A large number of observed cases may cause overdispersion (Breslow, 1984), i.e. if the assumption of a Poisson distribution is true, the unexplained variance is larger than expected, without any apparent misspecification of the model. Such results were obtained in several instances; the deviance of the full age-period-cohort model being considerably larger than the degrees of freedom. The overdispersion made it unsuitable to employ tests

*© 1999 Cancer Research Campaign*

based on the $\chi^2$ distribution. When we used the method suggested by Breslow (1984) to adjust for the overdispersion, it gave parameter estimates close to the standard maximum likelihood estimates of the Poisson model without this adjustment. Therefore, only risk estimates from the standard model are shown with standard errors adjusted for overdispersion. In the testing of different models, F-tests were performed to allow for the over-dispersion.

### RESULTS

### Cancer in situ

The reported annual incidence of cancer in situ in Sweden before 1960 was low, the age-standardized rate in 1958–1959 being about five cases per $10^5$. Following the introduction of screening, the reported incidence increased about 20-fold to 100 per $10^5$ in 1968. This level, sustained for a few years, declined slowly to about 90 per $10^5$ in the mid-1980s (Figure 1).

To describe the trend-wise development by age, we estimated separate models for periods before and after the introduction of widespread screening, i.e. 1958–1967 and 1968–1995. As shown in Table 1, the increasing trend during the 1958–1967 period was marked in all age-classes. The growth rate was fastest for women aged 20–24 years and diminished with increasing age. Starting in 1968 and thereafter, there were slight decreases in the reported incidence for young and middle-aged women (Table 1). The significant second-order terms for these groups reflect an initial increase up until the beginning of the 1980s for younger women and then a subsequent decrease (negative terms), while for middle-aged women it reflects a rapid decrease during the first few years after 1968 that gradually slowed down (positive terms).

Age-period-cohort modelling based on the period 1958–1992 using the Poisson distribution revealed that the age-period model was clearly superior to the age-cohort model, although the former was inferior to the full age-period-cohort model. The deviance of this model was much larger than the degrees of freedom, indicating either a poorly fitting model and/or overdispersion. An analysis confined to the period 1968–1992 still indicated the age-period model as the preferred one, since the age-period-cohort model was superior neither to the age-period model nor to the age-cohort model (Table 2). Considerable overdispersion still remained.

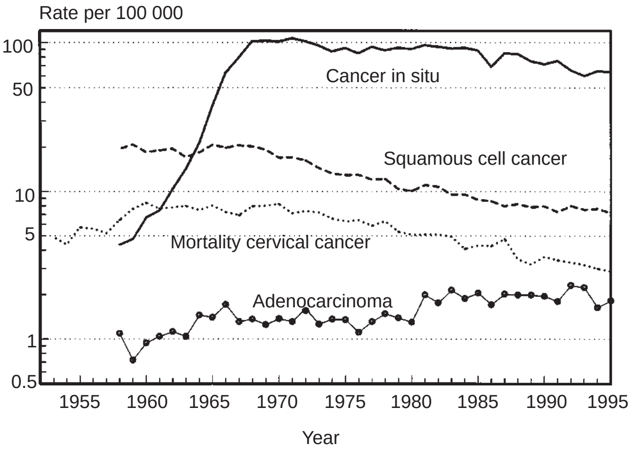

**Figure 1** Trends in age-standardized incidence rates of cancer in situ, squamous cell cancer and adenocarcinoma of the cervix uteri in Sweden, 1958–1995, obtained from the Cancer Registry (National Board of Health and Welfare, 1960–1998); and mortality rates from invasive cervical cancer in Sweden, 1953–1992, obtained from the Death Registry (National Board of Health and Welfare, 1956–1997)

### Squamous cell carcinoma

The age-standardized incidence of squamous cell carcinoma did not change significantly during the period 1958–1967. Only women aged 35–39 years had a growth rate significantly different from null, with an average annual decrease of 2.6% (Table 1).

**Table 1** Mean annual percentage change in age-specific and age-standardized incidences of reported cancer in situ (CIS), squamous cell cancer and adenocarcinoma of the cervix, 1958–1995, and in mortality from cancer of the cervix, 1953–1995, in Sweden[a]

| | CIS | | | Squamous cell cancer | | | Adenocarcinoma | | Mortality | | |
|---|---|---|---|---|---|---|---|---|---|---|---|
| | 1958–1967 | 1968–1995 | | 1958–1967 | 1968–1995 | | 1958–1995 | | 1953–1967 | 1968–1995 | |
| Age-class | % | % | Second Order term[c] | % | % | Second Order term[c] | % | Second Order term[c] | % | % | Second Order term[c] |
| 20–24 | 51.7*** | –1.3** | –*** | –4.7 | –1.9 | –** | *NA*[b] | | *NA*[b] | *NA*[b] | |
| 25–29 | 48.4*** | –0.1* | –*** | –5.3 | –1.7** | –*** | 3.1*** | – | *NA*[b] | *NA*[b] | |
| 30–34 | 45.3*** | –2.1*** | –*** | 0.8 | –1.5*** | – | 3.6*** | – | –0.8 | –3.6** | – |
| 35–39 | 39.9*** | –2.4*** | – | –2.6* | –2.7*** | +*** | 2.5*** | + | 2.7 | –3.0** | +* |
| 40–44 | 36.8*** | –2.4*** | + | 1.6 | –4.2*** | +** | 1.4 | – | 3.5* | –6.1*** | +* |
| 45–49 | 36.7*** | –2.6*** | +** | 1.1 | –5.5*** | +*** | 1.4 | + | 7.3*** | –7.0*** | + |
| 50–54 | 35.5*** | –1.2** | +*** | 1.7 | –5.5*** | +*** | 1.0 | – | 4.4** | –6.1*** | + |
| 55–59 | 31.7*** | –0.5 | +*** | –1.6 | –5.8*** | +*** | 1.1 | – | 4.3** | –6.2*** | – |
| 60–64 | 37.3*** | –0.9* | +* | –1.8 | –5.2*** | – | 1.8* | + | 3.8* | –4.1** | – |
| 65–69 | 21.5* | 0.7 | +* | 1.8 | –2.5*** | – | 2.1* | – | 2.0* | –2.7*** | –*** |
| 70–74 | 18.4** | 1.5* | +* | 1.6 | –2.1*** | + | 2.0* | – | 2.0 | –2.7*** | – |
| 75–79 | *NA*[b] | 2.6 | + | 4.0 | 0.6 | – | 0.3 | – | 5.6* | –1.6** | – |
| 80–84 | *NA*[b] | 0.1 | + | 4.1 | –1.7** | – | 1.3 | – | 3.2 | –0.8 | – |
| 85+ | *NA*[b] | *NA*[b] | | –0.2 | –0.1 | – | –0.4 | – | 1.6 | –0.2 | – |
| Age standardized | 40.7*** | –1.7*** | –** | 0.3 | –3.7*** | +*** | 1.8*** | – | 3.6*** | –4.0*** | – |

[a] **P* < 0.05, ***P* < 0.01, ****P* < 0.001; [b] too few observations; [c] sign + or– and *P*-value.

**Table 2** Results of fitting Poisson regression models to incidence of reported cancer in situ (CIS), 1968–1992, squamous cell cancer, 1968–1992, and adenocarcinoma, 1958–1992

| Model | CIS | | | Squamous cell cancer | | | Adenocarcinoma | | |
|---|---|---|---|---|---|---|---|---|---|
| | d.f | Deviance[a] | *P*-value[b] | d.f. | Deviance[a] | *P*-value[b] | d.f. | Deviance[a] | *P*-value[b] |
| Age | 52 | 2191.89 | | 52 | 1536.63 | | 78 | 221.47 | |
| Age+drift | 51 | 1204.12 | | 51 | 523.29 | | 77 | 126.89 | |
| Age+period | 48* | 1080.39 | 0.52 | 48 | 491.67 | <0.001 | 72* | 109.36 | 0.11 |
| Age+cohort | 36 | 890.35 | 0.14 | 36* | 115.26 | 0.13 | 60 | 91.41 | 0.03 |
| Age+period+cohort | 33 | 756.37 | | 33 | 97.23 | | 55 | 73.90 | |

d.f. Degrees of freedom; [a]Deviance from the standard Poisson model; [b]*P*-value based on test with F-statistic. Compares partial model with the full age-period-cohort model. The asterisk indicates the best fitting model used to estimate effects shown in Table 3.

**Table 3** Relative risks (RR) with 95% confidence intervals (CI) for incidence of reported cancer in situ (CIS), squamous cell cancer and adenocarcinoma; and for mortality from cervix cancer, for selected time periods and birth cohorts

| Determinant | CIS | | Squamous cell cancer | | Adenocarcinoma | | Mortality | |
|---|---|---|---|---|---|---|---|---|
| | RR | 95% CI | RR | 95% CI | RR | 95% CI | RR | 95% CI |
| Birth cohort[a] | | | | | | | | |
| 1903 | | | 2.07 | 1.70–2.51 | | | 1.72 | 1.48–2.00 |
| 1908 | | | 2.02 | 1.70–2.39 | | | 1.67 | 1.46–1.91 |
| 1913 | | | 1.86 | 1.60–2.16 | | | 1.58 | 1.39–1.78 |
| 1918 | | | 1.49 | 1.29–1.71 | | | 1.31 | 1.17–1.47 |
| 1923 | | | 1.00 | Reference | | | 1.00 | Reference |
| 1928 | | | 0.68 | 0.59–0.79 | | | 0.71 | 0.62–0.81 |
| 1933 | | | 0.45 | 0.38–0.53 | | | 0.47 | 0.40–0.55 |
| 1938 | | | 0.32 | 0.26–0.38 | | | 0.34 | 0.29–0.41 |
| 1943 | | | 0.27 | 0.23–0.33 | | | 0.21 | 0.17–0.27 |
| 1948 | | | 0.26 | 0.21–0.32 | | | 0.24 | 0.19–0.30 |
| 1953 | | | 0.26 | 0.21–0.33 | | | 0.21 | 0.16–0.29 |
| 1958 | | | 0.24 | 0.18–0.32 | | | 0.19 | 0.12–0.28 |
| 1963 | | | 0.22 | 0.15–0.32 | | | 0.20 | 0.11–0.36 |
| Time period | | | | | | | | |
| 1953–1957 | | | | | | | 0.67 | 0.60–0.75 |
| 1958–1962 | | | | | 0.72 | 0.57–0.90 | 1.01 | 0.91–1.11 |
| 1963–1967 | | | | | 1.01 | 0.83–1.24 | 1.00 | Reference |
| 1968–1972 | 1.00 | Reference | | | 1.00 | Reference | | |
| 1973–1977 | 0.88 | 0.80–0.96 | | | 0.92 | 0.75–1.13 | | |
| 1978–1982 | 0.89 | 0.81–0.98 | | | 1.16 | 0.95–1.41 | | |
| 1983–1987 | 0.82 | 0.75–0.90 | | | 1.42 | 1.18–1.71 | | |
| 1988–1992 | 0.71 | 0.64–0.79 | | | 1.45 | 1.20–1.74 | | |

[a]Central year of birth, e.g. 1898 refers to women born 1894–1902. All birth cohorts not shown. Estimates obtained from best fitting models, as indicated in Tables 2 and 4.

During 1968–1995, on the other hand, the age-standardized figure declined from 20 cases per $10^5$ in 1968 to around seven per $10^5$ in 1995 (Figure 1). This implied an average annual reduction of 3.7%, with marked differences between age-classes. The largest reduction occurred among middle-aged women, with an annual decrease of 4–6% for 40–64-year-old women. Among younger and older women the decrease gradually became lower, and in the youngest and oldest age-classes there was no significant average change (Table 1). The significant negative second-order terms in the two youngest age-classes reflect an initial increase in incidence and a later decrease from the mid 1980s. Among middle aged women the decline was rapid during the first few years after 1968 and then slowed down or ceased.

Age-period-cohort modelling of incidence data for squamous cell cancer during the entire period 1958–1992 did not produce an acceptable model fit. Systematic errors reflected a structural break following the introduction of screening. A modelling based on the period 1968–1992 produced more satisfactory results. Some overdispersion still occurred (deviance 97.23 on 33 degrees of freedom) and the age-cohort model was vastly superior to the age-period model (Table 2). The age-period-cohort model was not an improvement over the age-cohort model ($P = 0.13$).

We found a pronounced birth cohort pattern with little difference between the oldest cohorts followed by a monotonous decline among women born between 1913 and 1938 (Figure 2). The incidence relative to the reference birth cohort 1923 decreased from 1.86 in 1913 to 0.27 for the 1943 cohort. For younger cohorts the relative reduction in incidence remained fairly stable at 70–75%, without any evidence of increase in the youngest birth cohorts (Table 3). When we separated the birth cohort effects on various age classes (< 60, 60+; < 50, 50+; < 40, 40+; < 40, 40–60, 60+) virtually the same pattern over birth cohorts emerged. The steepest decline in relative risk was seen for ages 40–60 over the birth cohorts 1913–1943.

 © 1999 Cancer Research Campaign

**Table 4** Results of fitting Poisson regression models to mortality data for cervix cancer for different time periods

| | 1953–1967 | | | 1968–1992 | | |
|---|---|---|---|---|---|---|
| **Model** | **d.f.** | **Deviance[a]** | ***P*-value[b]** | **d.f.** | **Deviance[a]** | ***P*-value[b]** |
| Age | 26 | 162.31 | | 52 | 624.59 | |
| Age+drift | 25 | 80.80 | | 51 | 208.67 | |
| Age+period | 24* | 44.17 | 0.13 | 48 | 207.06 | <0.001 |
| Age+cohort | 12 | 47.17 | <0.001 | 36* | 43.67 | 0.83 |
| Age+period+cohort | 11 | 13.07 | | 33 | 42.53 | |

d.f. Degrees of freedom; [a]Deviance from the standard Poisson model [b] *P*-value based on test with F-statistic. Compares partial model with the full age-period-cohort model. The asterisk indicates the best fitting model used to estimate effects shown in Table 3.

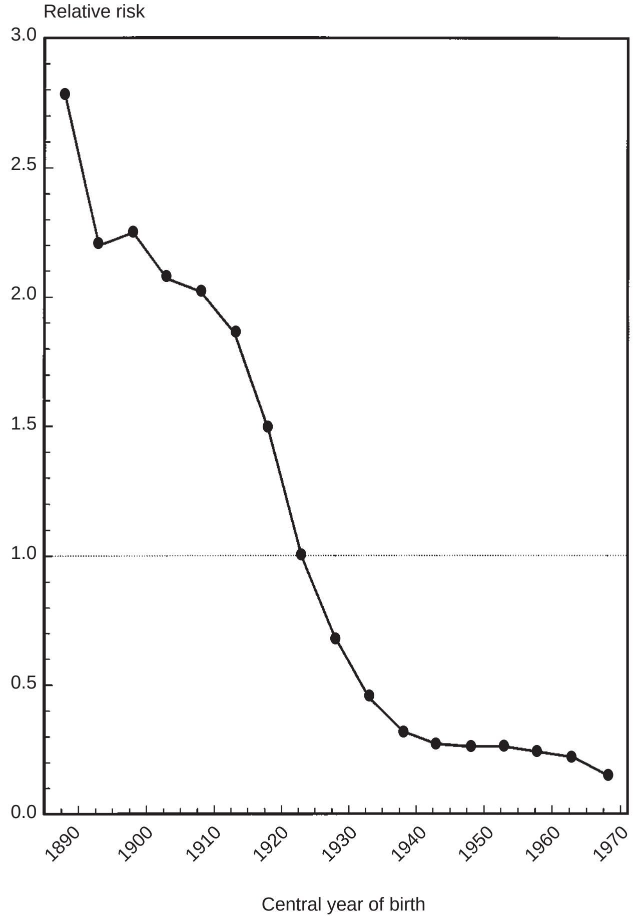

**Figure 2** Relative risks of squamous cell cancer incidence in Sweden by birth cohort according to the age-cohort model for the period 1968–1992, specified in Table 2

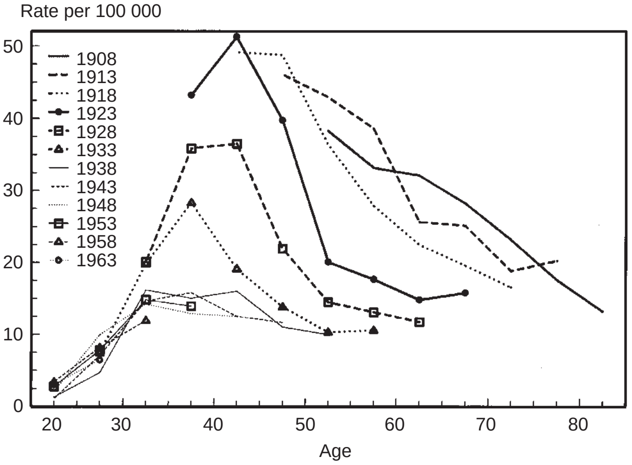

**Figure 3** Age-specific incidence of squamous cell cancer of the cervix uteri in Sweden 1958–1992, by birth cohort

Age-period-cohort modelling showed that there was not a great difference between the fits of the age-period and the age-cohort model (Table 2). Since the age-period-cohort model was significantly better than the age-cohort model, but not better than the age-period model, we chose the age-period model as a valid description of our data. The whole study period 1958–1992 could be used as the basis for a model that gave an acceptable fit to the data. We found a continuous increase in RR from the first to the last time period, with a doubling of the incidence over the 35 years of the study (Table 3).

### Adenocarcinoma

The age-standardized incidence of invasive adenocarcinoma increased substantially from 1958 to 1995 (Figure 1), on average by 1.8% annually. This growth was due to increases in incidence among women of all ages, most pronounced for ages 25–39. There were no significant second-order terms, indicating consistency in growth rates during the study period (Table 1). Adenocarcinomas accounted for 4.8% of all invasive cervical cancers in 1958 and for 19% in 1995.

### Mortality

During the 1950s the age-standardized mortality from cervical cancer increased from around four per $10^5$ in 1953 to around eight per $10^5$ in 1960. This level was maintained until about 1970, when a steady decrease started (Figure 1). In 1990 the age-standardized mortality rate was well under four per $10^5$, an average decrease of 4.0% annually from 1968 to 1995. The fastest decrease took place at ages 40–59 years, with a yearly average of 6–7%. At younger and older ages the decrease was slower, with no significant changes in the youngest and oldest age-classes. There were few significant second order terms, indicating limited changes in growth rates during the period 1968–1995 (Table 1).

Age-period-cohort modelling yielded more satisfactory results when the two time periods 1953–1967 and 1968–1992 were analysed separately rather than together. Two different patterns

© 1999 Cancer Research Campaign emerged (Table 4). The first time period could best be described by an age-period model, since the full age-period-cohort model was not an improvement over the age-period, but it was an improvement over the age-cohort model. For 1968–1992, the age-cohort model was an adequate representation of the data, since the full age-period-cohort model led to little further reduction in deviance and there was little evidence of overdispersion. For this time span the age-period model did not give a satisfactory representation of the data (Table 4).

In the period 1953–1967 the mortality rate was significantly lower (RR = 0.67) than that in the period 1963–1967. The birth cohort effects obtained from the age-cohort model during 1968–1992 were similar to those obtained for squamous cancer incidence (Table 3). There was little change among the oldest cohorts, while a rapid reduction in RR took place among birth cohorts born from 1913 to 1943, the RR of the latter being 0.21 with the 1923 cohort as reference. Among younger cohorts there was no further reduction, nor any evidence of increase in risk (Table 3). Estimation of birth cohort effects for mortality on the basis of restricted age classes produced a pattern similar to that for squamous cancer incidence.

### DISCUSSION

Our study covers a time period when an extensive prevention programme for cancer of the cervix was implemented in Sweden. This initiative followed successful efforts toward early detection and improved treatment of invasive cancer dating back to the first decades of this century (Pontén et al, 1995; Sparén et al, 1995). An organized programme (e.g. an active invitation to women for screening), coupled with an expansion of cytological laboratory services, were the means through which this intervention was carried out. However, there was also an awareness that opportunistic testing complemented the intervention programme; its expansion was indeed encouraged (National Board of Health and Welfare, 1970). In the 1970s and thereafter, when the screening programme was fully implemented, only about one-quarter of the smears were taken under the auspices of the organized screening programme, while the remaining three-quarters represented opportunistic screening, i.e. smears taken at hospitals, maternal health care units and other outpatient clinics (National Board of Health and Welfare, 1982; Pettersson et al, 1985; Gustafsson et al, 1995*b*).

The incidence and mortality pattern of cervical cancer was completely changed within a period of 10 years. After a substantial initial increase in the reported incidence of cancer in situ – though smaller for older women (Table 1) – the rates remained fairly stable thereafter. This indicates that the screening activities continued with full force (Figure 1). The overall incidence of and mortality from invasive cervical cancer decreased gradually, as reported earlier in Sweden (Pettersson et al, 1985; Gustafsson and Adami, 1990) and other countries (Hakama, 1982; Anderson et al, 1988; Hakama and Louhivuori, 1988; Levi et al, 1991; Sigurdsson, 1993).

The observed changes in incidence pattern were, however, not uniform for squamous cell cancer and adenocarcinoma. Trends in squamous cell cancer incidence could best be described as a cohort phenomena with three phases: while the oldest cohorts were not influenced by screening, women born between 1918 and 1938 experienced an increasingly large benefit and in more recent cohorts the reduction in incidence gradually levelled off (Figure 2). In fact, a reduction could be seen even among women born during the first 2 decades of this century (Figure 3). However, this could hardly be ascribed to the introduction of the screening programme in the mid-1960s, since these women were 50 years or older when large-scale screening started in Sweden. The reduction in incidence of squamous cell cancer caused a subsequent reduction of mortality for cervical cancer among the birth cohorts reflected (Table 3).

A different development was seen for adenocarcinomas of the cervix, namely a continuing increase in incidence affecting all ages, but particularly younger women. The relative risk, significantly lower during the first time period 1958–1962 compared to the reference period (1968–1972), levelled off between 1963 and 1977 and increased again through 1992 (Table 3). The incidence of adenocarcinoma seemed to be unaffected by screening, although screening might improve the prognosis of the disease due to detection at earlier stages (Sigurdsson, 1993). A recent study using SEER data, reported the increase of adenocarcinoma in US white women as a birth cohort phenomenon (Zheng et al, 1996). We could not find support for a cohort effect being more important than the period effect described above in the present data set. Other reports of increasing incidence of cervical cancer among US white women below the age of 50 are present (Larsen, 1994). No systematic review of histologies from the 1960s, to see if some of the old squamous cell tumours would now be classified as adenocarcinomas, was performed. To our knowledge there were no major changes in the classification of histology of cervical cancer tumours.

When interpreting the results of an age-period-cohort modelling, it is important to be aware of the fundamental problems caused by the linear dependence between the linear age, period and cohort effects. The non-linear effects, on the other hand, are uniquely defined, but a meaningful interpretation requires that the linear effects be included (Clayton and Schifflers, 1987*a*, 1987*b*; Holford, 1991; Tarone and Chu, 1996); for further discussion see Adami et al (1993*a*, 1993*b*). In none of the instances considered here (different histological categories and mortality) was the full age-period-cohort model a significant improvement over the best partial model. This meant that we did not try to find a solution to the identification problem of the full model, as the best partial model could be used. Contrary to what is sometimes believed, the fact that the age-cohort model contains more terms than the age-period model is not a problem, as the formal testing procedure takes account of the number of degrees of freedom.

A basic assumption underlying the age-period-cohort model is that the incidence rate depends multiplicatively on age, period and/or cohort. Among other things this means that in a model which includes cohort effects, the relative difference between birth cohorts is assumed to be the same at all ages. However, typically we only have observations for a given birth cohort over a limited number of age-classes. Thus, the oldest cohorts are only observed at old ages, while the most recent ones are only observed at young ages. This makes it difficult to judge whether the assumption of multiplicativity is reasonable. It also means that if the relative risk differences between cohorts were to change with age, the results of a cohort modelling might not be completely reliable. If, for example, the cohort effects are less pronounced at high ages, the cohort effects obtained from an age-cohort model may exaggerate

**Note** The raw data from this study have been placed on the Internet at the following website: www.grace.se/~shprsn/cervix.htm

 © 1999 Cancer Research Campaign the life-time reduction in risk for recent cohorts. When we separated the birth cohort effects on different age-classes, the overlapping of birth cohorts between ages was too small to make formal testing suitable. Since we cannot be sure that the assumption of multiplicativity between old and recent birth cohorts holds true, we need to interpret results about the magnitude of risk reduction with some caution.

The reduction in incidence of squamous cell cancer and mortality by 70–75% in more recent birth cohorts extends observations in previous studies (Pettersson et al, 1985; Gustafsson and Adami, 1990). We followed women born as late as the 1960s to elucidate if the positive effects of screening were maintained in younger birth cohorts. The levelling off of the risk for incidence of squamous cell cancer and mortality, starting from the 1943 and 1948 cohorts, respectively, indicates that the number of incident cases detected each year has entered a 'steady state' in the birth cohorts concerned.

If, as reported from the USA (Larsen, 1994; Weiss et al, 1994), Australia (Armstrong and Holman, 1981; Bourne and Grove, 1983) and Europe (Beral, 1984; Levi et al, 1989; Macgregor et al, 1994), there is an increasing trend of cervical cancer among younger women, possibly due to changes in sexual habits and increased transmission of human papilloma viruses, this trend would most likely also exist in Sweden. Our results indicate that screening so far has counteracted such a trend of squamous cell cancer, since we found no evidence of an increasing trend among younger birth cohorts. The high incidence of cervical cancer in women over 60 years of age in many screened populations today most likely depends on insufficient screening at younger ages. Thus, in the future one would expect reduced incidence rates of squamous cell cancer among older women as the cohorts that have been screened since age 20 or 30 become older (Figure 3). Intensified screening of older women does not necessarily reduce the incidence of squamous cell cancer. In a previous study we found strong indications that, apart from a lower prevalence of premalignant lesions at older ages, screening is less likely to detect precursors of cervical cancer among older women (Gustafsson et al, 1995*a*).

The continuous increase in the incidence of adenocarcinoma during the period of study indicates that the incidence of adenocarcinoma of the cervix is not affected by screening. This was also the conclusion of a recent case control study (Mitchell et al, 1995). Comparable increases have been reported in other populations (Bjørge et al, 1993; Kruger Kjaer and Brinton, 1993; Miller et al, 1993; Sigurdsson, 1993). The increase among women aged 25–39 years, although from a low level, is alarming. Adenocarcinoma of the cervix seems to entail a poorer prognosis than squamous cell cancers, also when controlling for clinical stage at diagnosis (Milsom and Friberg, 1983; Silcocks et al, 1987; Pengsaa et al, 1989; Hopkins and Morley, 1991; Sigurdsson et al, 1991; Kudo, 1992; Bjørge et al, 1993). It accounts for an increasingly higher proportion of the incident cervical cancers due to the underlying growth rate of the disease and the diminishing incidence of squamous cell cancer. The rapid growth rate of adenocarcinomas among younger women might contribute to the increasing incidence of cervical cancer among younger women recently reported.

In Sweden, a combination of organized and opportunistic screening seem to have reduced the incidence of squamous cell cancer in the most screened cohorts by around 70%. A prerequisite for this successful combination was the planned expansion of laboratory services and a strict follow-up of all abnormal tests, managed in part by the hospital laboratories themselves. In spite of this, the incidence of adenocarcinoma seems unaffected and is indeed increasing. New screening methods need to be developed to detect microinvasive adenocarcinoma as well as precursors of squamous cell cancer among older women.

### REFERENCES

Adami HO, Bergström R, Sparén P and Baron J (1993*a*) Increasing cancer risk in younger birth cohorts in Sweden (letter). *Lancet* **341**: 1409

Adami HO, Bergström R, Sparén P and Baron J (1993*b*) Increasing cancer risk in younger birth cohorts in Sweden. *Lancet* **341**: 773–777

Anderson GH, Boyes DA, Benedet JL, Le Riche JC, Matisic JP, Suen KC, Worth AJ, Millner A and Bennett OM (1988) Organisation and results of the cervical cytology screening programme in British Columbia, 1955–85. *Br Med J* **296**: 975–978

Armstrong B and Holman D (1981) Increasing mortality from cancer of the cervix in young Australian women. *Med J Aust* **9**: 460–462

Beral V (1984) Cancer of the cervix: a sexually transmitted infection? *Lancet* **1**: 1037–1040

Bjørge T, Thoresen SQ and Skare GB (1993) Incidence, survival and mortality in cervical cancer in Norwary, 1956–1990. *Eur J Cancer* **29A**: 2291–2297

Bourne RG and Grove WD (1983) Invasive carcinoma of the cervix in Queensland. Change in incidence and mortality, 1959–1980. *Med J Aust* **1**: 156–158

Breslow NE (1984) Extra-Poisson variation in log-linear models. *Appl Stat* **1**: 38–44

Brinton LA and Fraumeni JF (1986) Epidemiology of uterine cervical cancer. *J Chronic Dis* **39**: 1051–1065

Clayton D and Schifflers E (1987*a*) Models for temporal variation in cancer rates, I: age-period and age-cohort models. *Stat Med* **6**: 449–467

Clayton D and Schifflers E (1987*b*) Models for temporal variation in cancer rates, II: age-period-cohort models. *Stat Med* **6**: 469–481

Devesa SS, Young JL, Brinton LA, Fraumeni JF (1989) Recent trends in cervix uteri cancer. *Cancer* **64**: 2184–2190

Devesa SS, Blot WJ, Stone BJ, Miller BA, Tarone RE and Fraumeni JF (1995) Recent cancer trends in the United States. *J Natl Cancer Inst* **87**: 175–182

Farmery E and Gray JAM (1994) National Co-ordinating Network: Oxford

Gustafsson L and Adami HO (1990) Cytologic screening for cancer of the uterine cervix in Sweden evaluated by identification and simulation. *Br J Cancer* **61**: 903–908

Gustafsson L, Pontén J, Zack M and Adami HO (1997) International incidence rates of invasive cervical cancer after introduction of cytologic screening. *Cancer Causes Control* **8**: 755–763

Gustafsson L, Sparén P, Gustafsson M, Pettersson B, Wilander E, Bergström R and Adami HO (1995*a*) Low efficiency of cytologic screening for cancer in situ of the cervix in older women. *Int J Cancer* **63**: 804–809

Gustafsson L, Sparén P, Gustafsson M, Wilander E, Bergström R and Adami HO (1995b) Efficiency of organised and opportunistic cytological screening for cancer in situ of the cervix. *Br J Cancer* **72**: 498–505

Hakama M (1982) Trends in the incidence of cervical cancer in the Nordic countries. In: *Trends in Cancer Incidence – Causes and Practical Implications*. Magnus K (ed), pp. 279–292. New York Hemisphere: New York

Hakama M and Louhivuori K (1988) A screening program for cervical cancer that worked. *Cancer Surveys* **7**: 403–416

Holford T (1991) Understanding the effects of age, period, and cohort on incidence and mortality rates. *Annu Rev Public Health* **12**: 425–457

Hopkins MP and Morley GW (1991) A comparison of adenocarcinoma and squamous cell carcinoma of the cervix. *Obstet Gynecol* **7**: 912–917

Kruger Kjaer S and Brinton LA (1993) Adenocarcinomas of the uterine cervix: the epidemiology of an increasing problem. *Epidemiol Rev* **15**: 486–498

Kudo R (1992) Cervical adenocarcinoma. *Curr Top Pathol* **85**: 81–111

Larsen N (1994) Invasive cervical cancer rising in young white females (news). *J Natl Cancer Inst* **86**: 6–7

Levi F, La Vecchia C, Te VC and Gutswiller F (1989) Incidence of invasive cervical cancer in the Swiss canton of Vaud, and a note on screening. *J Epidemiol Community Health* **43**: 121–124

Levi F, La Vecchia C, Randriamiharisoa A and Boyle P (1991) Cancer mortality in young adults in Switzerland, 1951–1989. *J Cancer Res Clin Oncol* **117**: 497–501

Macgregor JE, Campbell MK, Mann EMF and Swanson KY (1994) Screening for cervical intraepithelial neoplasia in north east Scotland shows fall in incidence and mortality from invasive cancer with concomitant rise in preinvasive disease. *Br Med J* **308**: 1407–1411

*© 1999 Cancer Research Campaign*

Mattsson B and Wallgren A (1984) Completeness of the Swedish Cancer Register. Non-notified cancer cases recorded on death certificates in 1978. *Acta Radiol Oncol* **23**: 305–313

Miller BE, Flax SH, Arheart K and Photopulos G (1993) The presentation of adenocarcinoma of the uterine cervix. *Cancer* **72**: 1281–1285

Milsom I and Friberg LG (1983) Primary adenocarcinoma of the uterine cervix. A clinical study. *Cancer* **52**: 942–947

Mitchell H, Medley G, Gordon I and Giles G (1995) Cervical cytology reported as negative and risk of adenocarcinoma of the cervix: no strong evidence of benefit. *Br J Cancer* **71**: 894–897

National Board of Health and Welfare (1956–1997) *Causes of Death 1953–1995*. Statistics Sweden: Stockholm

National Board of Health and Welfare (1960–1998) *Cancer Incidence in Sweden 1958–1995*. National Board of Health and Welfare, The Cancer Registry: Stockholm

National Board of Health and Welfare (1970) *Gynecological Mass Examinations 1967 and 1968. The Campaign and Its Results (in Swedish with English summary)*. National Board of Health and Welfare: Stockholm

National Board of Health and Welfare (1976) *Statistical Reports HS 1976. Gynecological Mass Screening in Sweden*. Official Statistics of Sweden. Statistics Sweden: Stockholm

National Board of Health and Welfare (1982) *Principles and Routines for Gynecological Health Examinations. Report From an Expert Group of the National Board of Health and Welfare (in Swedish)*. National Board of Health and Welfare: Stockholm

National Board of Health and Welfare (1984) *SOSF 1984:32*. National Board of Health and Welfare: Stockholm

Parkin DM, Pisani P and Ferlay J (1993) Estimates of the worldwide incidence of 18 major cancers in 1985. *Int J Cancer* **55**: 891–903

Pengsaa P, Udomthavornsuk B, Vatanasapt W, Pesi M, Tungvorapongchai V and Shibata Y (1989) Survival analysis of cervical cancer patients at Sinagarind Hospital 1976–1987. *J Med Assoc Thai* **72**: 346–350

Pettersson F, Björkholm E and Näslund I (1985) Evaluation of screening for cervical cancer in Sweden: trends in incidence and mortality 1958–1980. *Int J Epidemiol* **14**: 521–527

Pisani P, Parkin DM and Ferlay J (1993) Estimates of the wordwide mortality from eighteen major cancers in 1985. *Int J Cancer* **55**: 891–903

Pontén J, Adami HO, Bergström R, Dillner J, Friberg LG, Gustafsson L, Miller AB, Parkin M, Sparén P and Trichopoulos D (1995) Strategies for global control of cervical cancer. *Int J Cancer* **60**: 1–26

Quinn M, Babb P, Jones J and Allen E (1999) Effect of screening on incidence of and mortality from cancer of cervix in England: evaluation based on routinely collected statistics. *Br Med J* **318**: 904–908

Sasieni P (1991) Trends in cervical cancer mortality (letter). *Lancet* **Sep 28**: 818–819

Sasieni P, Cuzick J and Farmery E (1995) Accelerated decline in cervical cancer mortality in England and Wales (letter). *Lancet* **346**: 1566–1567

Sasienie P and Adams J (1999) Effect of screening on cervical cancer mortality in England and Wales: analysis of trends with an age period cohort model. *Br Med J* **318**: 1244–1245

Sigurdsson K (1993) Effect of organized screening on the risk of cervical cancer. Evaluation of screening activity in Iceland 1964–1991. *Int J Cancer* **54**: 563–570

Sigurdsson K, Hrafnkelsson J, Geirsson G, Gudmundsson J and Salvarsdóttir A (1991) Screening as a prognostic factor in cervical cancer: analyses of survival and prognostic factors based on Icelandic population data. *Gynecol Oncol* **43**: 64–70

Silcocks PB, Thornton JH and Murphy M (1987) Squamous and adenocarcinoma of the uterine cervix: a comparison using routine data. *Br J Cancer* **55**: 321–325

Sparén P, Gustafsson L, Friberg LG, Pontén J, Bergström R and Adami HO (1995) Improved control of invasive cervical cancer in Sweden over six decades by earlier clinical detection and better treatment. *J Clin Oncol* **3**: 715–725

Tarone RE and Chu KC (1996) Evaluation of birth cohort patterns in population disease rates. *Am J Epidemiol* **143**: 85–91

Villard L, Murphy M and Vessey MP (1989) Cervical cancer deaths in young women (letter). *Lancet* **Feb 18**: 377

Weiss LK, Kau TY, Sparks BT and Swanson GM (1994) Trends in cervical cancer incidence among young black and white women in metropolitan Detroit. *Cancer* **73**: 1849–1854

Zheng T, Holford TR, Zheng M, Chen Y, Liu W, Ward BA and Boyle P (1996) The continuing increase in adenocarcinoma of the uterine cervix: a birth cohort phenomenon. *Int J Epidemiol* **25**: 252–258

 *© 1999 Cancer Research Campaign*
